# Thermally conductive polyamide 6/carbon filler composites based on a hybrid filler system

**DOI:** 10.1088/1468-6996/16/6/065001

**Published:** 2015-11-05

**Authors:** Sung Min Ha, O Hwan Kwon, Yu Gyeong Oh, Yong Seok Kim, Sung-Goo Lee, Jong Chan Won, Kwang Soo Cho, Byoung Gak Kim, Youngjae Yoo

**Affiliations:** 1 Division of Advanced Materials, Korea Research Institute of Chemical Technology, Daejeon 34114, Korea; 2 Department of Polymer Science and Engineering, Kyungpook National University, Daegu 41566, Korea; 3 Department of Chemical Convergence Materials and Nanomaterials Science and Engineering, University of Science and Technology, Daejeon 34113, Korea

**Keywords:** polymer composites, thermal conductivity, thermography, modeling

## Abstract

We explored the use of a hybrid filler consisting of graphite nanoplatelets (GNPs) and single walled carbon nanotubes (SWCNTs) in a polyamide 6 (PA 6) matrix. The composites containing PA 6, powdered GNP, and SWCNT were melt-processed and the effect of filler content in the single filler and hybrid filler systems on the thermal conductivity of the composites was examined. The thermal diffusivities of the composites were measured by the standard laser flash method. Composites containing the hybrid filler system showed enhanced thermal conductivity with values as high as 8.8 W (m · K)^−1^, which is a 35-fold increase compared to the thermal conductivity of pure PA 6. Thermographic images of heat conduction and heat release behaviors were consistent with the thermal conductivity results, and showed rapid temperature jumps and drops, respectively, for the composites. A composite model based on the Lewis–Nielsen theory was developed to treat GNP and SWCNT as two separate types of fillers. Two approaches, the additive and multiplicative approaches, give rather good quantitative agreement between the predicted values of thermal conductivity and those measured experimentally.

## 1. Introduction

Polymer matrix composites are commonly used in several industries, owing to their remarkable properties such as corrosion resistance, durability, miniaturization capabilities, weight reduction capabilities, and low fabrication cost. In addition, with the increase in demand for high power output and faster circuits in electronic devices, the choice of heat-release material in electronic packaging is becoming increasingly important. A rapid increase in the heat density of the product is not only detrimental to the performance of the electronic equipment, but also causes malfunctioning and decrease in the durability of the device [1, 2]. Polymeric materials generally have low thermal conductivity in the range of 0.1–0.5 W (m · K)^−1^. Consequently, polymers need to be filled with thermally conductive fillers, such as carbon, ceramic, and metallic fillers, to prevent heat loss and achieve high thermal conductivity.

Many reports are available in the literature on thermally conductive polymer composites prepared using various matrix materials such as polypropylene [3], polyamide 6 (PA 6) [4], polyamide 66 [5], liquid crystal polymers [6] and epoxy [7]. Further, the thermal conductivities of the composites have been shown to increase by incorporating various thermally conductive fillers such as graphite, graphene, carbon nanotubes, boron nitride, and alumium nitride [8–12]. In addition, many studies currently show that the thermal conductivity of the composites can be significantly enhanced by using hybrid filler systems [13–17].

The thermal diffusivity of composites can be determined by the laser flash method and the thermal conductivity (K, W (m · K)^−1^) can be calculated using equation (1):

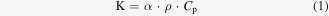
where *α* is the thermal diffusivity (mm^2^ s^−1^), *ρ* is the density (g cm^−3^), and *C*_p_ is the specific heat (J (g · K)^−1^). Thermal diffusivity is the rate of phonon conduction through the material during the heating and cooling of the material. In the laser flash method, a heat pulse of short duration is incident on the front side of the sample, and the increase in the temperature of the sample is recorded on the back side. Thermal diffusivity is calculated using the half maximum temperature, dimension of sample, and the half time [18–20].

The purpose of this work is to examine the enhancement in thermal conductivity and changes in annealing behavior obtained by incorporating graphite nanoplatelets (GNP) and single walled carbon nanotubes (SWCNTs) as filler materials in the PA 6 matrix. The two filler materials considered have very different shapes; GNP has a platelet shape, whereas SWCNT has a fiber-like shape.

## 2. Experimental details

### 2.1. Materials

Commercial grade PA 6, EN 200 (KP Chemtech Co., Korea), was used in this study. PA 6 has a glass transition temperature and melting temperature of 55 and 224 °C, respectively. The two kinds of thermally conductive carbon fillers, namely GNP and SWCNT, were purchased from Timcal Co. (Switzerland) and Nanocyl Co. (Belgium), respectively. GNP generally has a particle size of 80 *μ*m and the SWCNT used in this work had a diameter of 2 nm and length of 1–5 *μ*m. Two types of SWCNTs, namely SWCNTs without any treatment (referred to as pristine SWCNTs, hereafter) and with carboxyl group treatment (referred to as f-SWCNTs, hereafter), were used to investigate the effect of filler dispersion on the properties of the composites. The properties of the materials are shown in table 1.

**Table 1.** Properties of materials used in the study.

Material	Commercial designation	Specifications	Supplier
*Polymer*			
Polyamide 6 (PA 6)	EN 200	Relative viscosity: 2.5,	KP Chemtech Co., Korea
		Ash content: 0.07%,	
		T_g_: 55 °C	
		T_m_: 224 °C	
		Thermal conductivity—0.25 W (m · K)^−1^	
*Fillers*			
Graphite nanoplatelet (GNP)	C-Therm	Bulk density: 0.18 g cm^−3^	Timcal Co., Switzerland
		(Expanded graphite: 0.005 ∼ 0.01 g cm^−3^)	
		Average size: 80 *μ*m	
		Thermal conductivity—400 W (m · K)^−1^	
Single walled carbon nanotube (SWCNT)	NC 1100	Pristine SWCNT	Nanocyl Co., Belgium
	NC 1101	Functionalized SWCNT	
		- Thermal conductivity—3000 W (m · K)^−1^	
		- Carbon purity > 70%—	
		- Average diameter: 2 nm	
		- Specific surface area > 1000 m^2^ g^−1^	

### 2.2. Preparation of composites

Before preparation of the polymer composites, PA 6, GNP, and SWCNT were dried for a minimum of 24 h in a vacuum oven at 80 °C. All the composites were prepared in a DSM Xplore micro-compounder, which has two co-rotating conical screws and a barrel capacity of 15 mL, using a screw speed of 80 rpm and a barrel temperature of 260–270 °C. The test specimens were fabricated using a DSM Xplore micro-injection molding machine with the barrel temperature at 270 °C, mold temperature at 80 °C, and injection molding pressure at 16 bar. The dimensions of the mold specimen used for thermal conductivity measurements were 10 × 10 × 50 mm^3^ and the injection molded specimens were immediately placed in a vacuum desiccator for a minimum of 24 h prior to testing. The formulations used for preparing the (PA 6/GNP)/SWCNT composites are shown in table 2. The contents of additional SWCNT were varied from 0 to 5 wt% based on 100% PA 6/GNP composition.

**Table 2.** Formulations used for preparing the (PA 6/GNP)/SNCWT composites.

Sample	Compositions	PA6/GNP contents (wt% ratio)	SWCNT contents (wt%)
1	PA6	100/0	0
2	PA6/GNP	85/15	0
3	PA6/GNP	70/30	0
4	PA6/GNP	50/50	0
5, 11	(PA6/GNP)/SWCNT, (PA6/GNP)/f-SWCNT	85/15	1
6, 12	(PA6/GNP)/SWCNT, (PA6/GNP)/f-SWCNT	70/30	1
7, 13	(PA6/GNP)/SWCNT, (PA6/GNP)/f-SWCNT	50/50	1
8, 14	(PA6/GNP)/SWCNT, (PA6/GNP)/f-SWCNT	85/15	5
9, 15	(PA6/GNP)/SWCNT, (PA6/GNP)/f-SWCNT	70/30	5
10, 16	(PA6/GNP)/SWCNT, (PA6/GNP)/f-SWCNT	50/50	5

### 2.3. Characterization

The thermal diffusivities of the composites were measured at room temperature using a Netzsch laser flash thermal diffusivity apparatus (LFA 447 NanoFlash). Test specimens 2.5 × 8 × 8 mm^2^ in size were cut out the center part of the injection-molded specimens. The density and specific heat were measured using a Micromeritics Gas Pycnometer (Accupyc 1330) and TA instruments modulated differential scanning calorimeter (MDSC Q200), respectively, and used to calculate thermal conductivity [21]. For the density and specific heat measurements, the test samples were prepared as powders using a SPEX sample freezer/mill 6750, which were dehydrated for 24 h in a desiccator. Annealing tests were performed for the composites in a vacuum oven at 150 °C for various durations of time. After annealing, the thermal diffusivities of the test specimens were measured immediately. Fractured surface of the composites were studied by scanning electron microscopy (SEM, Tescan Mira 3 LMU FEG, 20 kV). Thermal imaging was also performed using an FLIR infrared camera (T300).

## 3. Results and discussion

### 3.1. Morphology

**Figure 1.**
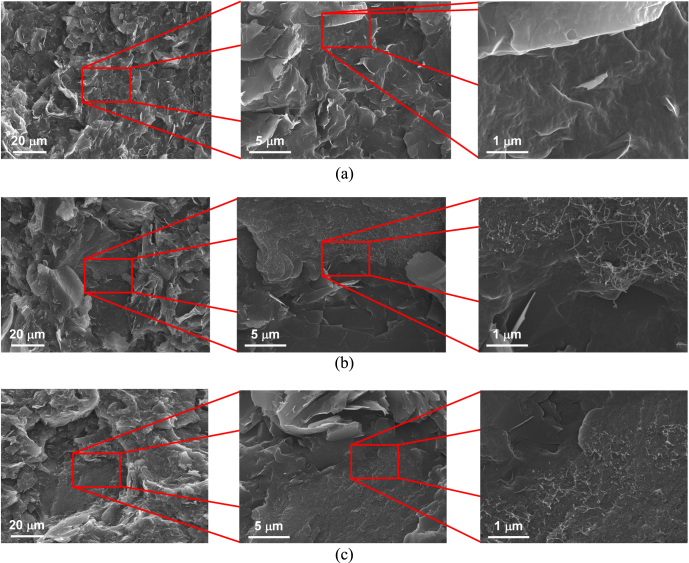
SEM micrographs of (a) PA 6/GNP composites, (b) (PA 6/GNP)/SWCNT composites, and (c) (PA6/GNP)/f-SWCNT composites.

**Figure 2.**
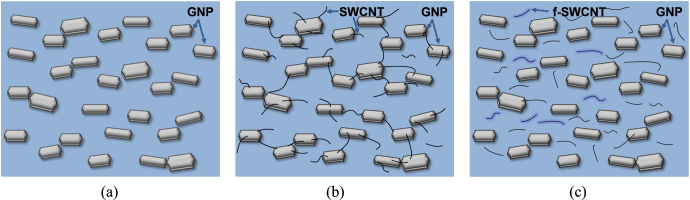
Schematic illustration of the morphology of (a) PA 6/GNP composites, (b) (PA 6/GNP)/SWCNT composites, and (c) (PA6/GNP)/f-SWCNT composites.

SEM micrographs of the PA 6/GNP, (PA 6/GNP)/SWCNT, and (PA 6/GNP)/f-SWCNT composites are shown in figure 1. A representative SEM image of the PA 6/GNP composite is shown in figure 1(a). As shown in the figure, in this case, the GNP particles are well-dispersed in the PA 6 matrix. Phonon transport is facile in the large particles of GNP and the limited number of interfaces (at which the phonons are scattered) results in high thermal conductivity of the PA 6/GNP composite. In the case of the composite containing pristine SWCNTs, aggregated SWCNTs are observed with revealing the pulled-out morphology, as shown in figure 1(b). Aggregated SWCNTs, which have much higher thermal conductivity than GNP, can act as very efficient phonon transporting media because of facile physical contact among SWCNTs. As a result, the thermal conductivity of the composite is significantly increased upon adding pristine SWCNTs. However, when f-SWCNTs are added, the morphology of the composite is significantly altered due to inherent affinity of f-SWCNTs towards the PA 6 matrix. As evident from figure 1(c), the f-SWCNTs are well-dispersed in the PA 6 matrix. In this case, phonon transport is rapid in both the GNP and f-SWCNT particles. However, the considerably increased number of interfaces caused by the uniformly dispersed f-SWCNTs act as phonon scattering sites, as a result of which the composites containing f-SWCNTs show lower thermal conductivity compared to those containing pristine SWCNTs. Figure 2 shows a schematic illustration of the morphological changes in the PA 6/GNP, (PA 6/GNP)/SWCNT, and (PA 6/GNP)/f-SNCWT composites that occur upon the addition of SWCNTs and f-SWCNTs. This diagram summarizes the collective observations made from many SEM micrographs.

### 3.2. Thermal conductivity

**Figure 3.**
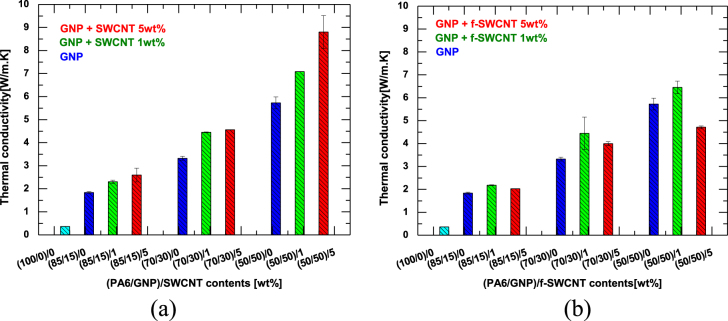
(a) Thermal conductivity of PA 6/GNP and (PA 6/GNP)/SWCNT composites and (b) thermal conductivity of PA 6/GNP, (PA6/GNP)/f-SWCNT composites.

Figure 3(a) shows the effect of the composition of the hybrid filler system on the thermal conductivity of the composite. The thermal conductivities of the composites increase with increasing GNP content. For example, the thermal conductivity increases from 0.363 W (m · K)^−1^ for pure PA 6, to 1.594, 3.525, and 5.607 W (m · K)^−1^ for PA 6 containing 15, 30, and 50 wt% of GNPs, respectively. Furthermore, for a given GNP content, the thermal conductivity gradually increases upon increasing the SWCNT content. This increase is attributed to the increase in the thermal pathway as a result of the formation of a network structure between the GNPs and SWCNTs [10, 14, 22]. However, there is significant difference between the thermal conductivity of composites containing pristine SWCNT and f-SWCNT. The thermal conductivities of the composites increase with increasing SWCNT content in the case of pristine SWCNT. However, in the case of f-SWCNT, the thermal conductivity of the composite containing 5 wt% of f-SWCNT is lower than that with 1 wt% of f-SWCNT, as shown in figure 3(b). Since the f-SWCNT has better dispersion in the matrix compared to pristine SWCNT, the number of interfaces formed with the dispersed SWCNT increases and an effective thermal conducting network structure is not formed. It is expected that the interfacial interaction and interfacial thermal resistance between the PA6 and SWCNT were affected depending upon the treatment of the SWCNT [14]. Another possible reason for the lower thermal conductivity of the composites based on f-SWCNT than those from SWCNT is the intrinsic low thermal conductivity of f-SWCNT owing to the possible defects originating during the modification process.

### 3.3. Thermal annealing

**Figure 4.**
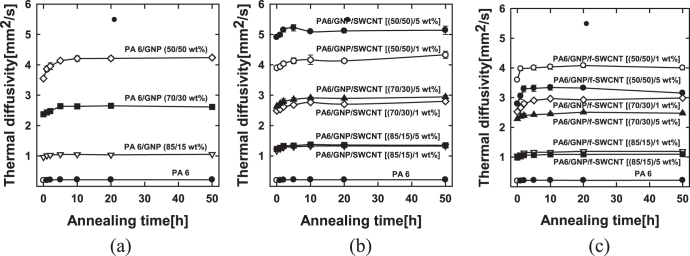
Thermal diffusivity of the specimen as a function of annealing time (a) PA6/GNP composites, (b) (PA6/GNP)/SWCNT composites, and (c) (PA6/GNP)/f-SWCNT composites.

Annealing tests were performed to confirm the long-term stability of the thermally conductive polymer composites. The specimens used for the thermal diffusivity measurements were annealed for 1, 2, 5, 10, 20 and 50 h in a vacuum oven at 150 °C. As shown in figure 4, the thermal diffusivity of the composites increases up to an annealing duration of 10 h, beyond which the thermal diffusivity stays constant, for all the samples (i.e., both PA 6/GNP composites as well as composites with the hybrid filler system). The hybrid filler system does not significantly affect the thermal conductivity of the annealed composites. However, composites with high filler loading over 30 wt% exhibit increased changes in the thermal diffusivity during thermal annealing compared to pure PA 6 and composites with low filler loading. This is attributed to the relaxation process that the polymer chains and fillers, which are forced to be oriented along the flow direction during the polymer melt-processing, undergo during annealing.

### 3.4. Modeling

There are many factors such as thermal conductivity, modulus, aspect ratio, orientation, filler shape, and volume fraction of the filler that affect the thermal conductivity of the composites. Various models have been proposed to predict the effective thermal conductivity of polymer composites [6, 23–25]. To model the properties of composites and to correlate the experimental data with composite models, many assumptions are required. For example, the polymer matrix is not affected by the presence of the filler, e.g., no change in crystallinity, the filler is perfectly aligned, there is good adhesion between the matrix and the filler, the matrix and the filler are isotropic, and there are no filler-filler interactions or agglomerations. Additionally, differences in the morphology of real composites and injection molded specimens including a skin–core structure are also important. However, in the simplified composite model, the properties of the matrix and fillers, filler shape and orientation and filler volume fraction only, are considered. Several of these models available in the literature are summarized below and the predictions from these models were compared with our experimental results.

The most basic thermal conductivity models for composite materials are the standard rule of mixtures (equation (2)), inverse rule of mixtures (equation (3)), and geometric rule of mixtures (equation (4)) [26]

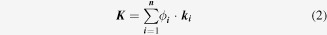


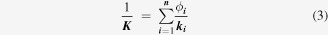


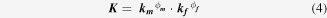
where *K* and *k_i_* are the thermal conductivities of the composite and the *i*th component, respectively, whereas *φ_i_* is the volume fraction of the *i*th constituent. Besides these three basic models, a number of other thermal conductivity models have been proposed in the literature and are summarized below.

The Maxwell–Eucken theoretical model describes the thermal conductivity of composites consisting of randomly dispersed particles in a polymer matrix. This model is used to predict the conductivity of a system containing spherical, non-interacting particles in a continuous matrix and is given by equation (5) [27]

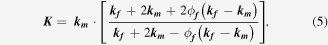



The Bruggeman model was created using different assumptions for permeability and field strength compared to the Maxwell–Eucken model and can be expressed by equation (6) [27, 28]

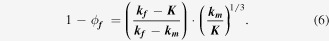



For filaments of uniform cross-sectional area arranged in parallel, the Halpin–Tsai model is used between in-plane field equations and boundary conditions to the transverse transport coefficient. This model also considers the shape of the filler particles and is given by equation (7)

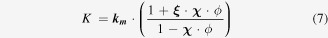
where 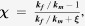 
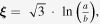
 and *a* and *b* are the width and thickness of the fillers, respectively [29].

The Lewis and Nielsen model (equation (8)) is similar to the Halpin–Tsai model and accounts for the shape and orientation of the filler particles, as well as the packing fraction

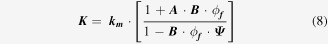
where 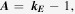 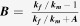, 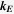 is the Einstein coefficient, and 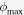 is the maximum packing fraction of the filler. 
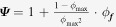
 in the Lewis and Nielsen model. In the modified Lewis and Nielson model, 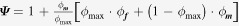 [23, 30–33].

**Figure 5.**
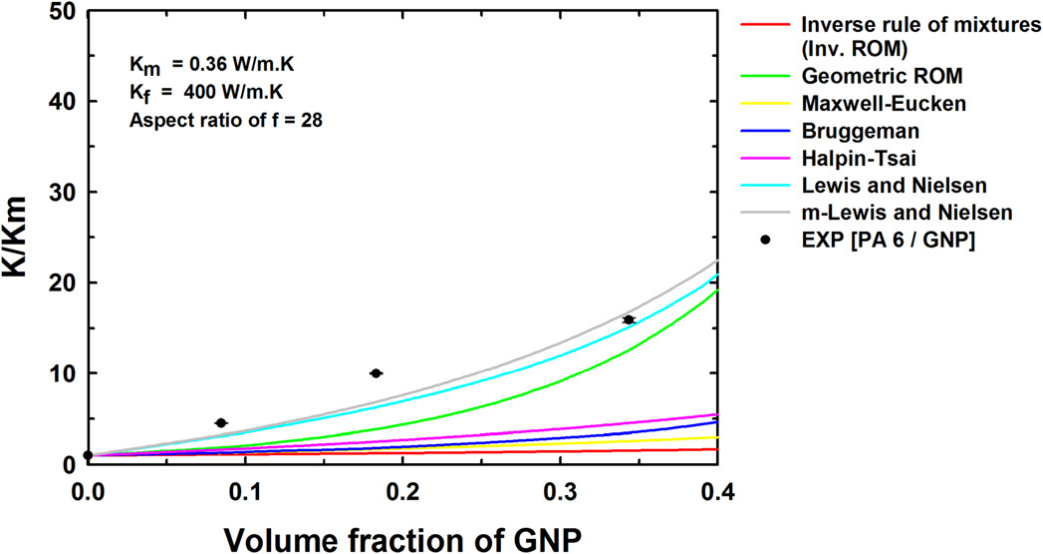
Experimental data and theoretical predictions of the thermal conductivity of PA 6/GNP composites obtained using various models.

Figure 5 shows the theoretical predictions and experimental thermal conductivity results for the PA 6/GNP composites. The experimental results obtained were fitted to each of the theoretical models presented above. Since most of the theoretical predictions do not account for the high stresses that occur during melt-processing, the thermal conductivity is underestimated in most of the models as evident from the figure. Among the various models, the Lewis and Nielsen and modified Lewis and Nielsen models are in relatively good agreement with the experimental results.

In the theoretical models mentioned above, the predictions are based on the population of a single filler in the matrix. However, the hybrid filler system composites considered in this study are composed of two fillers, namely GNPs and SWCNTs, and a model that accounts for the two fillers is needed to predict the experimental results properly [34, 35]. Two-population models can be constructed using an additive approach or a multiplicative approach. In the additive approach (equation (9)), the individual contributions of the two fillers are summed without double counting the contribution of the matrix

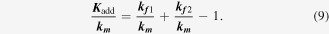



**Figure 6.**
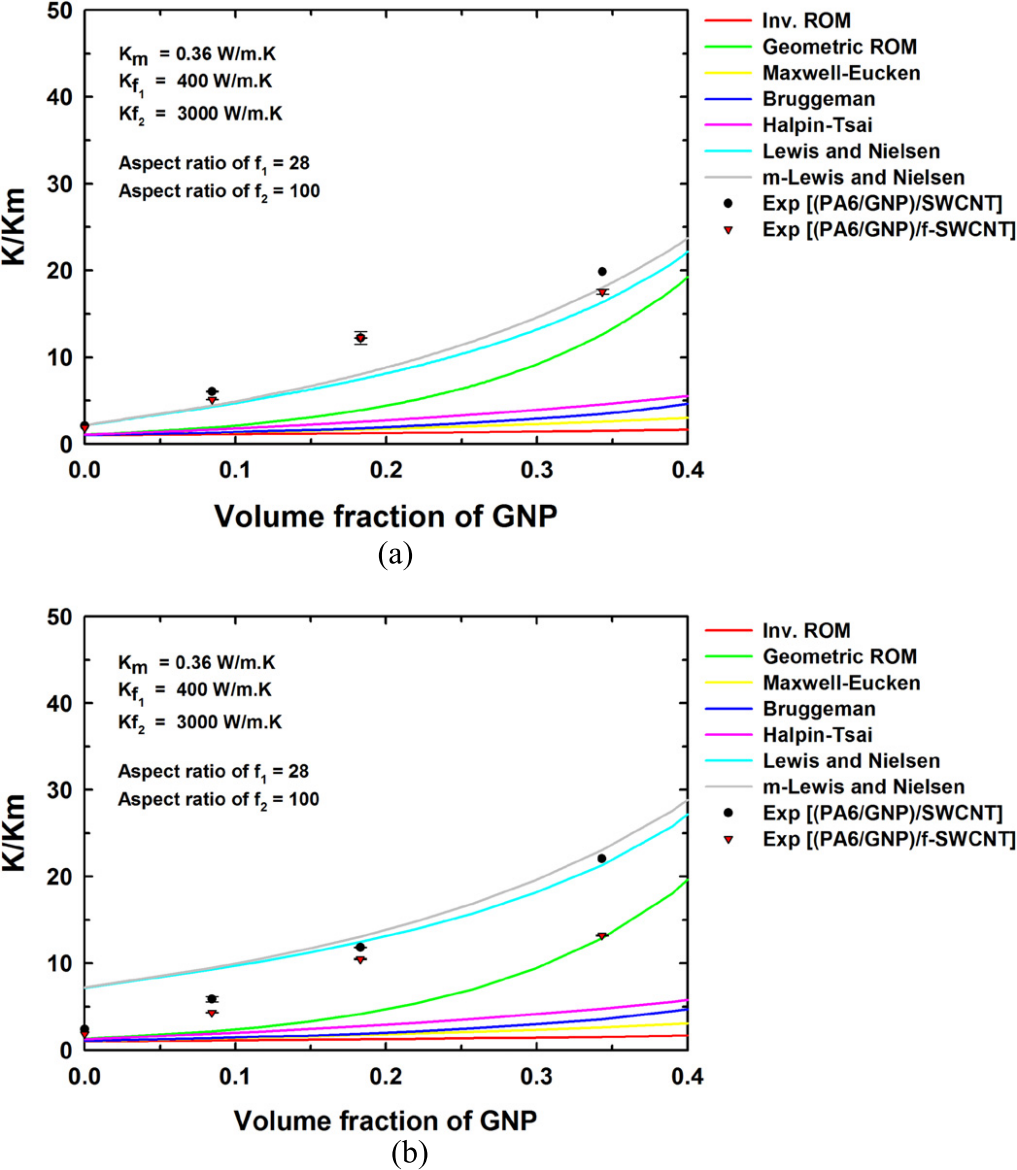
Experimental data and theoretical thermal conductivity predictions obtained using various models by the additive approach for (a) (PA 6/GNP)/SWCNT (1 wt%) and (b) (PA 6/GNP)/SWCNT (5 wt%).

**Figure 7.**
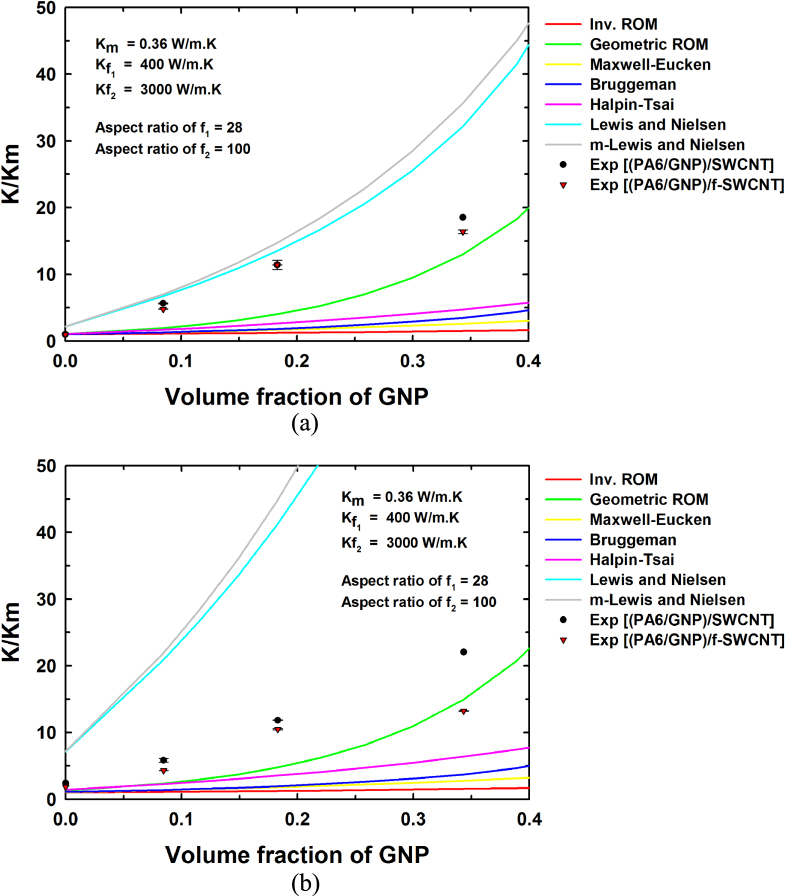
Experimental values and theoretical thermal conductivity predictions obtained using various models by the multiplicative approach for (a) (PA 6/GNP)/SWCNT (1 wt%) and (b) (PA 6/GNP)/SWCNT (5 wt%).

In the above equation, 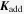 is the thermal conductivity of the hybrid filler composite predicted using the additive approach and 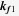 and 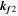 are the thermal conductivities of the composites containing GNPs and SWCNTs, respectively. On the other hand, in the multiplicative approach, the contribution of the composite containing SWCNTs is calculated first and this composite is then considered to be the matrix for calculating the GNP contribution. In other words, the contribution of the GNPs towards thermal conductivity is calculated by considering the PA 6/SWCNT composite ( 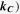 as the matrix rather than the neat polymer matrix ( 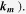 The contributions of the fillers are multiplied as given by equation (10)

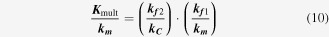
where 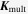 is the thermal conductivity of the composite predicted using a multiplicative approach. Figures 6 and 7 show the results from the two-population models for the thermal conductivity of the composites containing hybrid filler systems for two SWCNT concentrations, namely 1 wt% and 5 wt%. Both the additive and multiplicative two-population models predict similar improvements in thermal conductivity, although the multiplicative approach predicts a larger thermal conductivity improvement with increase in the filler content compared to the additive approach for both cases. This is attributed to the fact that the multiplicative approach treats the PA6/SWCNT composite as the matrix for the GNP addition. Figures 6(a) and 7(a) show nearly similar curves for the composites at low filler contents. Similarly, figures 6(b) and 7(b) show nearly similar curves at low filler contents. At higher filler contents, the predicted thermal conductivity values are somewhat higher, owing to the differences in the assumed and actual aspect ratios and particle orientations.

### 3.5. Thermography

**Figure 8.**
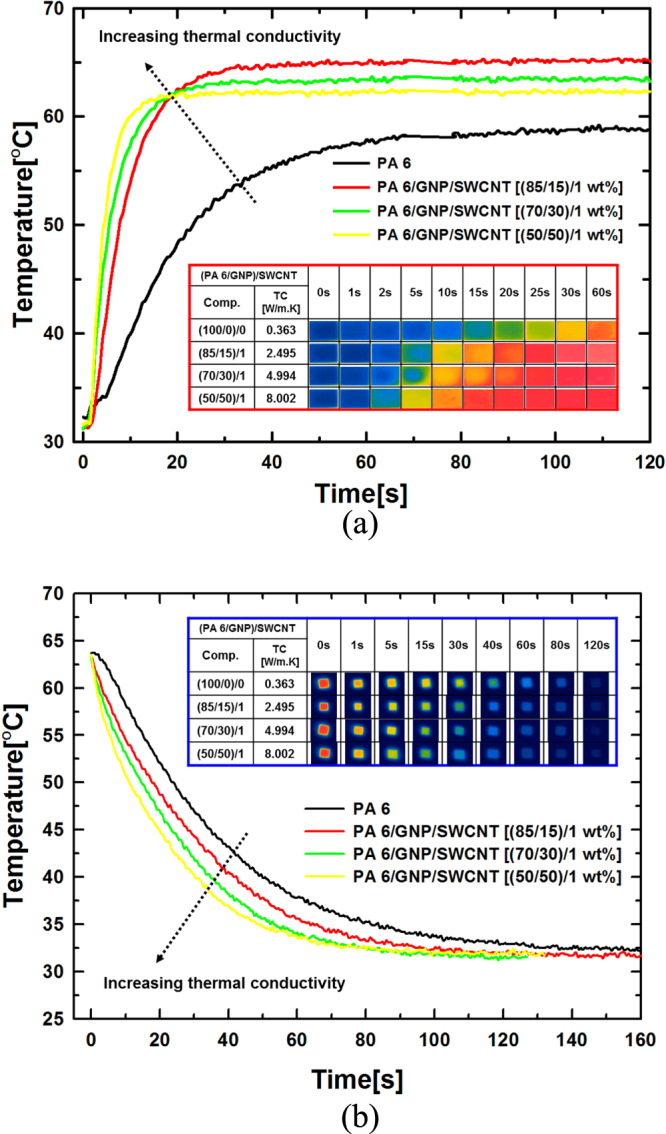
Thermographic images of the heat conduction behavior of (PA 6/GNP)/SWCNT composites (temperature set point: 70 °C).

Figure 8 shows the thermographic images of the composites. The thermographic images were acquired using an infrared camera by capturing the thermal images of the samples placed on a hot-plate whose surface temperature was set at 70 °C. Two modes of temperature changes, namely heat conduction and heat release by the specimen, were considered and the surface temperature changes of the specimen were measured as a function of elapsed time. The pure PA 6 sample exhibits a slow change in surface color, which is characteristic of a typical thermal insulator. On the other hand, as the thermal conductivities of the composite samples increase, the surface color of the composites changes rapidly with time. Additionally, time to reach the maximum or minimum temperature of the composite surface is faster along with higher thermal conductivity. The maximum temperature of composite surface exhibits a different tendency in the heat conduction because of the heat loss by the air. The thermography results show good agreement with the thermal conductivity values shown in figure 3 [36].

## 4. Conclusions

Thermally conductive composites based on PA6, GNP, and SWCNT were prepared by melt-processing using a DSM Xplore micro-compounder. Thermal conductivities were calculated using the experimentally measured thermal diffusivity, density, and specific heat values. The thermal conductivities of the composites were found to increase with an increase in the GNP content. The composites containing pristine SWCNTs showed increased thermal conductivity with an increase in the SWCNT content while composites containing f-SWCNTs showed at first increased and then decreased thermal conductivity behavior. It appears that the addition of pristine SWCNTs to the PA 6 matrix can be an effective way to increase the thermal conductivity of the composites, owing to the aggregation of SWCNTs, which results in efficient phonon transport. However, when functionalized SWCNTs are added, the resulting composites show low thermal conductivity, owing to the increase in the number of interfaces between the PA 6 matrix and carbon fillers. Defect sites, which are possibly created during the modification process, can be another reason for intrinsic low thermal conductivity of functionalized SWCNT. Upon adding a combination of 50 wt% of GNP and 5 wt% of pristine SWCNT to the PA 6 matrix, the thermal conductivity increased to 8.8 W (m · K)^−1^, which is an approximately 35-fold increase compared to the corresponding value for pure PA6. The thermal conductivity results and the surface colors of the composite specimens were consistent with the thermographic images. In addition, various models were compared to predict the changes in the thermal conductivity of the composites. The Lewis and Nielsen model and modified Lewis and Nielsen model show the best fit with experimental results. To consider the contribution of the two different fillers, a two-population model was introduced. The additive and multiplicative approaches were discussed and it was found that the additive approach shows better prediction of the conductivity of the composites while the multiplicative approach shows a serious overestimation of the thermal conductivity.
